# Anomaly-Detection-Driven Screening of Thermodynamic Stability from Composition Descriptors Alone

**DOI:** 10.1021/acs.jpclett.5c03772

**Published:** 2026-02-10

**Authors:** Keisuke Makino, Yudai Yamaguchi, Naoto Tanibata, Hayami Takeda, Ryo Kobayashi, Masayuki Karasuyama, Masanobu Nakayama

**Affiliations:** † Department of Advanced Ceramics, 12982 Nagoya Institute of Technology, Nagoya, Aichi 466-8555, Japan; ‡ Department of Physical Science and Engineering, 12982 Nagoya Institute of Technology, Nagoya, Aichi 466-8555, Japan; § Department of Computer Science, 12982 Nagoya Institute of Technology, Nagoya, Aichi 466-8555, Japan

## Abstract

Materials informatics tends to rely on existing structural database searches that constrain exploration by omitting unregistered compositions. In this study, an autoencoder-based anomaly detector was developed using composition-only descriptors as input features. The model was trained on thermodynamically stable phases—defined as those on the convex hull with an energy above hull (Δ*E*_*hull*_) of 0 eV/atom—as well as nearly stable phases with Δ*E*_*hull*_ < 0.01 eV/atom, sourced from the Materials Project inorganic database. The reconstruction error (RMSE) was used as the anomaly score. It was shown that the RMSE increased systematically with apparent thermal destabilization—that is, increasing energy above hull. It was also shown that for 50,000 dummy oxides with an intentionally perturbed charge balance, the RMSE increased in proportion to the magnitude of the total charge imbalance, indicating that departures from charge neutrality could be captured even without explicit charge information. Feature-importance analysis suggested that element pairs (two-element combinations) were the principal factors governing the reconstruction RMSE. Accordingly, for 50,000 charge-compensated virtual oxides, we ordinally encoded the valence-shell type as s = 1, p = 2, d = 3, and f = 4 and defined, for each element pair, a coarse indicator given by the product of the two codes (hereafter, the spdf product). For each pair, we evaluated the correspondence between the median RMSE (taken over all compositions containing that pair) and its spdf product and obtained an approximately monotonic relationship—that is, pairs with smaller spdf products tended to have a lower median RMSE, whereas those with larger spdf products tended to have higher values. By contrast, pairs containing Ta consistently deviated downward from this relationship (i.e., exhibited a lower RMSE), suggesting that not only the spdf product but other descriptor information could also influence the assessment of synthesizability.

## Graphical abstract

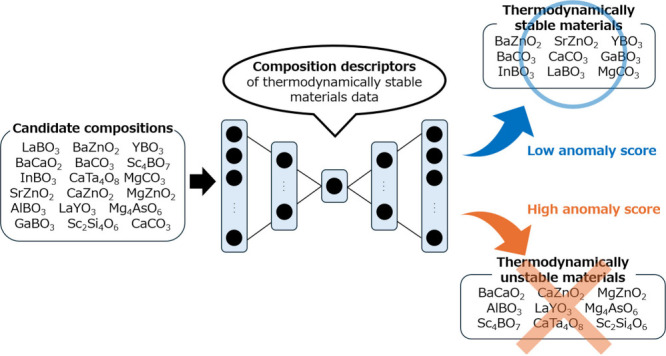

Materials informatics (MI), which combines information science with materials science, has garnered considerable attention in recent years as a means of accelerating research and development. Numerous MI studies have reported the optimization and discovery of lithium-ion battery materials, which are indispensable power sources for portable devices and electric vehicles.^1–3^ These MI efforts have largely relied on in-database searches,^4–8^ wherein candidate compounds that satisfy target properties have been narrowed down from hundreds of thousands of crystal structures registered in existing databases—including the Inorganic Crystal Structure Database (ICSD),^9^ Materials Project,^10–12^ Crystallography Open Database (COD),^13–18^ and computationally optimized structure databases, such as the Materials Project,^10–12^ AFLOW,^19,20^ and Open Quantum Materials Database (OQMD).^21,22^

However, this approach has a fundamental limitation in that materials with compositions or structures that are not yet registered in these databases fall outside the search space. Indeed, Villars and co-workers^23^ estimated that, even for ternary inorganic compounds, only approximately 16% have been discovered to date; moreover, for quaternary and higher systems, the fraction falls below 1%, indicating that a vast proportion of the potential chemical space remains unexplored. Discovered in 2011, Li_10_GeP_2_S_12_ ^24^—a solid electrolyte material that has attracted attention as a battery material—exhibits exceptionally high lithium-ion conductivity; however, it was not included in the inorganic crystal structure databases. Clearly, the discovery and evaluation of materials not listed in such databases has the potential to yield breakthroughs in functional materials. To reveal this unexplored domain, it is essential to virtually generate candidate materials from multicomponent compositions outside existing databases that can be considered to be synthesizable.

As an example of prior efforts to address the above problem, previous studies have evaluated the material phase stability based solely on compositional information.^25–27^ These studies successfully predicted the phase stability of a given chemical composition with statistical significance. In particular, the features could be constructed by transforming the elemental characteristics (atomic number, electronegativity, ionic radius, etc.) into statistical descriptors—including the mean, maximum, minimum, and similar aggregates. However, because these works relied on classification analysis, they required not only inputs corresponding to synthesizable materials (positive examples), but also information on unsynthesizable materials (negative examples). However, databases based on experimental data (e.g., ICSD^9^ and JCPDS-ICDD^28,29^ ) register only synthesized materials (positive examples), rendering the above approach difficult to apply. Although databases derived from materials simulations can register data for hypothetical materials and thereby provide, to some extent, inputs that can serve as negative examples, there remains the problem that the registered crystal structures are not guaranteed to be optimal for the corresponding compositions.

Methodologies have recently been proposed to generate plausible crystal structures for a given composition. Examples include methods based on metaheuristics and diffusion models—such as USPEX and MatterGen—which are also used in image generation applications.^30–32^ Although crystal-structure determination for inorganic solids has long been a major challenge, and these methods are highly intriguing, the structures proposed from a single composition are not unique; many candidate structures are typically generated, necessitating nontrivial computational resources for validation. Moreover, the evaluation of complex multicomponent compositions has become technically challenging, making these methods less suitable for scanning a large number of compositions. However, methodologies that perform systematic evaluations with considerable computational resources have recently been proven effective. Merchant et al.^33^ computationally discovered 2.2 million structures that lie below the convex hull by applying partial substitution to existing structures^34^ and *ab initio* random structure searching from the composition.^35^ However, evaluating the properties of hypothetical materials individually using materials simulation requires significant computational resources.^8^ Taken together, these developments suggest that over the past decade, various attempts have been made to push exploration technologies for materials that are absent from databases toward practical solutions. Simultaneously, MI studies that have introduced an anomaly detection perspective have begun to appear. For example, PhaseSelect evaluates unknownness using the reconstruction error from an autoencoder coupled with attention-based representation learning from element-set-only information, thereby proposing unexplored phase fields.^36^ In another study targeting NASICON-type cathodes, a crystal graph convolutional neural network (CGCNN) model that ingests structural descriptors was combined with positive-unlabeled (PU) learning to perform a binary classification of synthesizability.^37^ However, the former remains at the level of chemical novelty of element sets and does not directly address thermal stability, whereas the latter presupposes a large volume of density functional theory (DFT)-derived labeled data and explicit negative examples.

Additionally, several synthesizability predictors using the Materials Project have been proposed based on PU learning frameworks—including SynthNN^38^ and SynCoTrain.^39^ These methods can formulate synthesizability as a supervised or PU-based binary classification problem, and therefore require defining an unlabeled pool and/or constructing pseudonegative examples; in practice, the learned decision boundary can be sensitive to the collection or generation of such unlabeled/pseudonegative data. Moreover, the SynCoTrain^39^ model has been demonstrated primarily in a structure-aware setting, whereas evaluations using composition-derived descriptors alone have not been performed. In this context, recent studies have explored anomaly detection-based measures of unknownness and PU learning-based synthesizability classification; however, the use of only positive examples—that is, compounds that are considered highly synthesizable—to train a composition-descriptor-based anomaly detection model for assessing thermodynamic stability has rarely been explored.

In this study, we focused on anomaly detection, a machine-learning method, under the conditions that (i) tens of thousands of inorganic compounds registered in crystal databases consisted, in principle, of only positive examples (although some data for hypothetical materials existed that could serve as negatives), and (ii) synthesizability should be judged rapidly to enable broad compositional screening. In anomaly detection, the model is trained only on positive data, learns the patterns of positives, and then identifies the inputs that do not conform to these patterns as anomalies (negatives). Specifically, we extracted materials with small energy above hull^5^ as positive synthesizable materials and trained a deep-learning autoencoder.^40^ We hypothesized that for patterns derived from positive data, the autoencoder’s input and output would coincide, whereas for negative data, they would not. Using a descriptor set constructed from the composition, we built a system that—by employing the reconstruction error (RMSE) between the input and output as an anomaly score—could instantaneously score synthesizability when a candidate material with an arbitrary composition was input. Notably, the proposed framework did not use any structural descriptors—such as bond lengths or lattice parameters—instead, all evaluations were performed solely within the descriptor space derived from the composition. Moreover, the reconstruction RMSE was interpreted as a measure of the proximity to the compositional characteristics learned from a stable training data set. Here, we present several validation examples and discuss the effectiveness of anomaly detection-based exploration in the discovery of new materials.

In this study, we evaluated the performance of an anomaly detection autoencoder with composition descriptors as inputs under the following conditions: from the April 2025 release of the Materials Project, we used all 58,235 entries of registered compounds whose energy above hull was <0.01 eV/atom. The Materials Project was selected because it provides a large, energy-labeled data set suitable for quantitative assessment of composition-based anomaly detection. When multiple polymorphs existed for the same chemical composition, only the structure with the lowest energy above hull was retained, with the others being excluded. This polymorph filtering was introduced only to avoid duplicate entries at the composition level and did not imply that the model ingested or depended on the structural information. The input vector consisted of 1994-dimensional histogrammed^41^ descriptors built from elemental attributes, including the atomic number, group number, period number, Mendeleev number, atomic weight, melting point in the metallic state, electronegativity, atomic/ionic/crystal/covalent radii, and numbers of s, p, d, and f electrons. Here, we introduce descriptors that connect the characteristics originating from the coexistence of the two elements. The procedure and parameters used to histogram these descriptors are described in Section S1 of the Supporting Information.

Figure 1(a) shows a diagnostic plot of the reconstruction error for the test data obtained using an autoencoder. A clear trend is evident in which the coefficient of determination increases as the latent dimension increases. In the model with 16 latent dimensionality adopted in this study, *R* ^2^ exceeded 0.95, indicating that the descriptors were reconstructed with high accuracy. Increasing the latent dimensionality to 32 resulted in only marginal improvement, with the *R* ^2^ rising to 0.96. Accordingly, we employed a neural network model with a latent dimensionality of 16.

**Figure 1:**
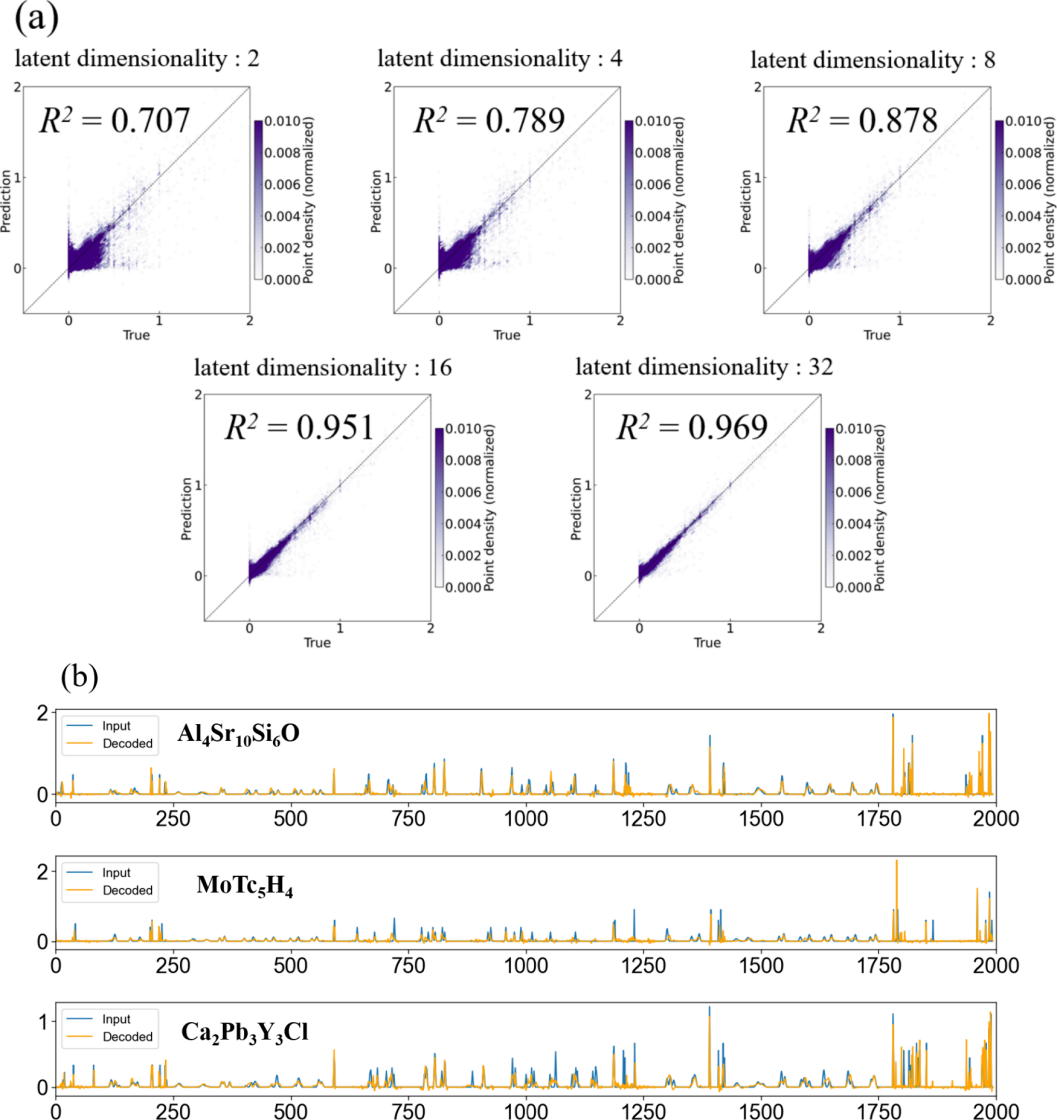
Reconstruction accuracy of the input descriptors by the autoencoder. (a) Diagnostic plot of the test data with respect to the reconstruction error of the autoencoder for different latent dimensionalities (input dimensionality: 1994). The color of each point represents the local point density estimated by Gaussian kernel density estimation (KDE), and the color bar shows density values normalized to 0–0.01. Higher values (darker colors) indicate higher point densities. (b) Comparison between the input descriptors (blue) and reconstructed descriptors (orange) for the three samples that exhibited the largest reconstruction errors when passed through the autoencoder with a latent dimensionality of 16.

Figure 1(b) compares the input and reconstructed descriptors for the three samples that exhibited the largest reconstruction errors when passed through this model with a latent dimensionality of 16. Even in these high-error cases, the overall profiles agreed reasonably well, suggesting that the autoencoder sufficiently captured the characteristics of the input data. From these results, we could conclude that the autoencoder appropriately learned the features of the input data and proved to be a valid anomaly detection system.

Figure 2 shows changes in the RMSE distribution when the compounds from the Materials Project were grouped by the energy above hull, which is a metric of thermal stability. The range 0.00–0.01 eV/atom corresponds to the region included in the training data set. As the energy above hull increased, the RMSE distribution shifted continuously toward higher values, clearly indicating a tendency for synthesizability to decrease. Accordingly, we could conclude that the model appropriately learned the compositional characteristics of thermally stable materials with low energy above hull. Because the RMSE distributions partially overlapped across the stability bins, we further quantified the screening performance by receiver operating characteristic (ROC) analysis using the Materials Project entries, where compounds with *E*_*hull*_ < 0.01 eV/atom and >0.01 eV/atom were treated as positive and negative classes, respectively, and the RMSE (lower is more stable) was used as the score. The resulting ROC curve yielded an area under the curve (AUC) of 0.710 (Figure S2), indicating that the RMSE provided moderate discrimination between stable and unstable regions but did not perfectly separate them, consistent with the overlap observed in Figure 2.

**Figure 2:**
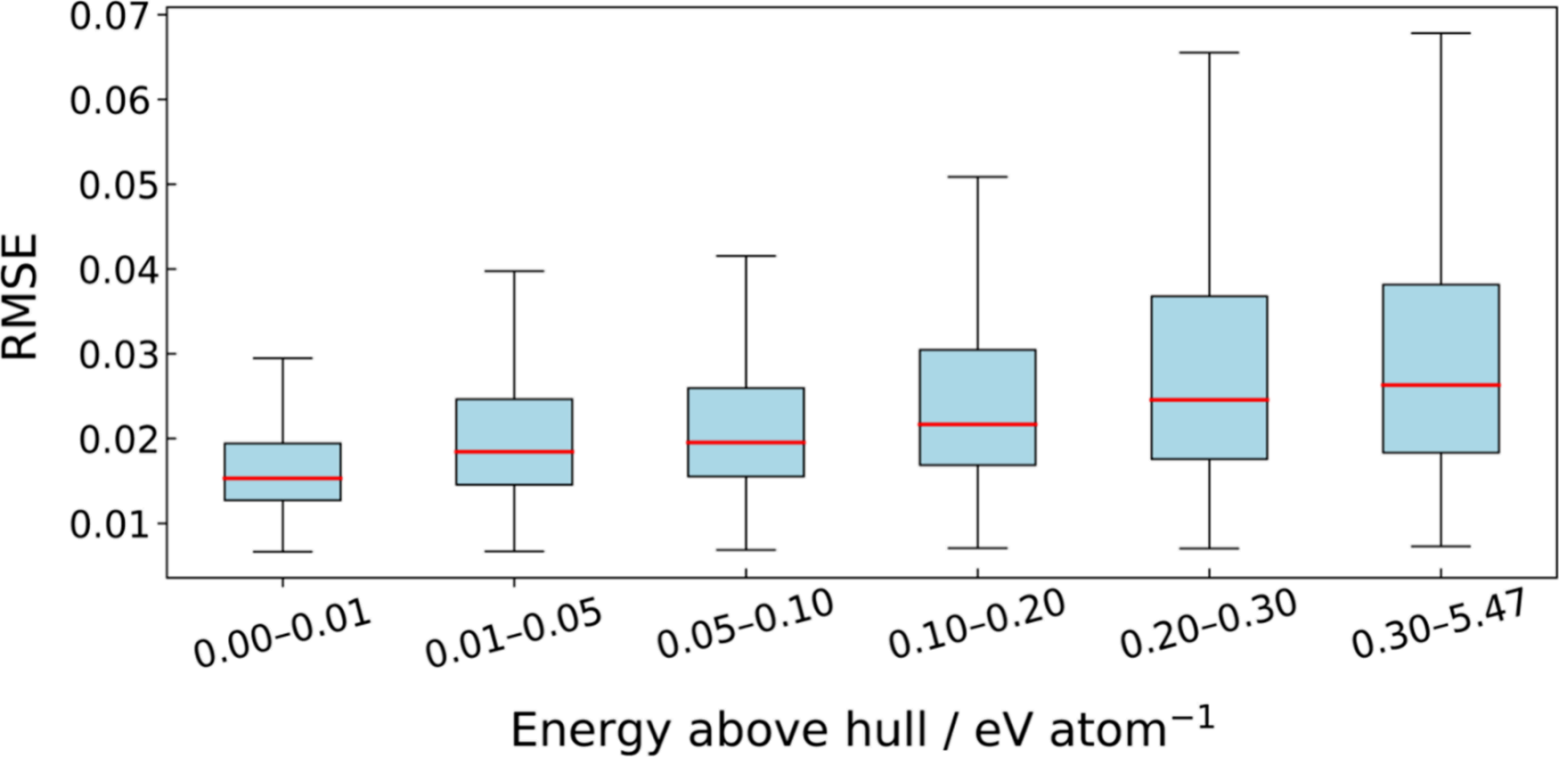
Changes in the RMSE distribution for compounds in the Materials Project when grouped by energy above hull. The boxes indicate the interquartile range (Q1–Q3), and the red line within each box denotes the median. The whiskers extend to 1.5 × the interquartile range (IQR), and outliers beyond this range are omitted.

We also examined all materials in the Materials Project whose energy above hull (*E*_*hull*_) > 0.01 eV/atom and were therefore regarded as thermodynamically unstable. This data set, *E*_*hull*_ > 0.01, contained 62,247 entries in total, of which 15,093 materials (24.2%) were linked to ICSD data sets that have reported syntheses. According to ref.,^42^ among the approximately 243,000 registered entries, 19,077 structures were identified as being derived from DFT calculations at that time. Thus, the majority of the ICSD data set originated from experimental sources, and the materials contained therein could be considered to be experimentally synthesizable. We then focused only on the top 50 materials with the lowest-RMSE values among 15,093 suggested compounds (listed in Table S5 in the Supporting Information), 23 of them (46%) had reported syntheses (verified by manual inspection thorough the ICSD^9^ reference information). Even taking 24.2% as the true proportion in the population, the probability that 24 or more of the top-50 data set would have reported syntheses was very small (approximately 6.3 × 10^–4^ using a one-sided exact binomial test), which indicates that the synthesized materials were statistically significantly enriched in the low-RMSE region. Moreover, this top-50 data set included materials for which syntheses had been reported despite having relatively high energy above hull values (such as 0.44 and 0.95 eV). Consequently, it was likely that the proposed model did not simply classify materials with a small energy above hull as normal but rather preferentially assigned them to the normal class of materials whose compositional features resembled those of the training data. In other words, despite deviating from the database stability label (e.g., high *E*_*hull*_), compounds that remained close to the learned stable composition distribution could still be prioritized by low RMSE, which could help identify potentially hidden synthesizable candidates.

Next, we examined the relationship between the total charge per atom and the RMSE. The Materials Project contains numerous oxides, which consist of cations and oxide ions, which therefore must satisfy charge neutrality. Accordingly, we generated 50,000 oxide compositions without enforcing charge neutrality, input them into the anomaly detection model, and computed the RMSE. The virtual composition combined elements whose oxidation states were typically well-defined, specifically main-group elements and transition metals, in d^0^/d^10^ configurations: Mg^2+^, Ca^2+^, Sr^2+^, Ba^2+^, Sc^3+^, Y^3+^, La^3+^, Ti^4+^, Zr^4+^, Hf^4+^, Nb^5+^, Ta^5+^, Zn^2+^, B^3+^, Al^3+^, Ga^3+^, In^3+^, C^4+^, Si^4+^, Ge^4+^, Sn^4+^, P^5+^, As^5+^, Sb^5+^, and S^6+^. Of course, cases exist in which the assumed oxidation state differs (for example, NbO_2_, SiO, and SnO); however, we can assume that such instances are rare. As some generated formulas may incidentally satisfy charge neutrality, we excluded compositions whose total charge fell within the + threshold and evaluated the RMSE distribution for the remainder, the results of which are shown in Figure 3.

**Figure 3:**
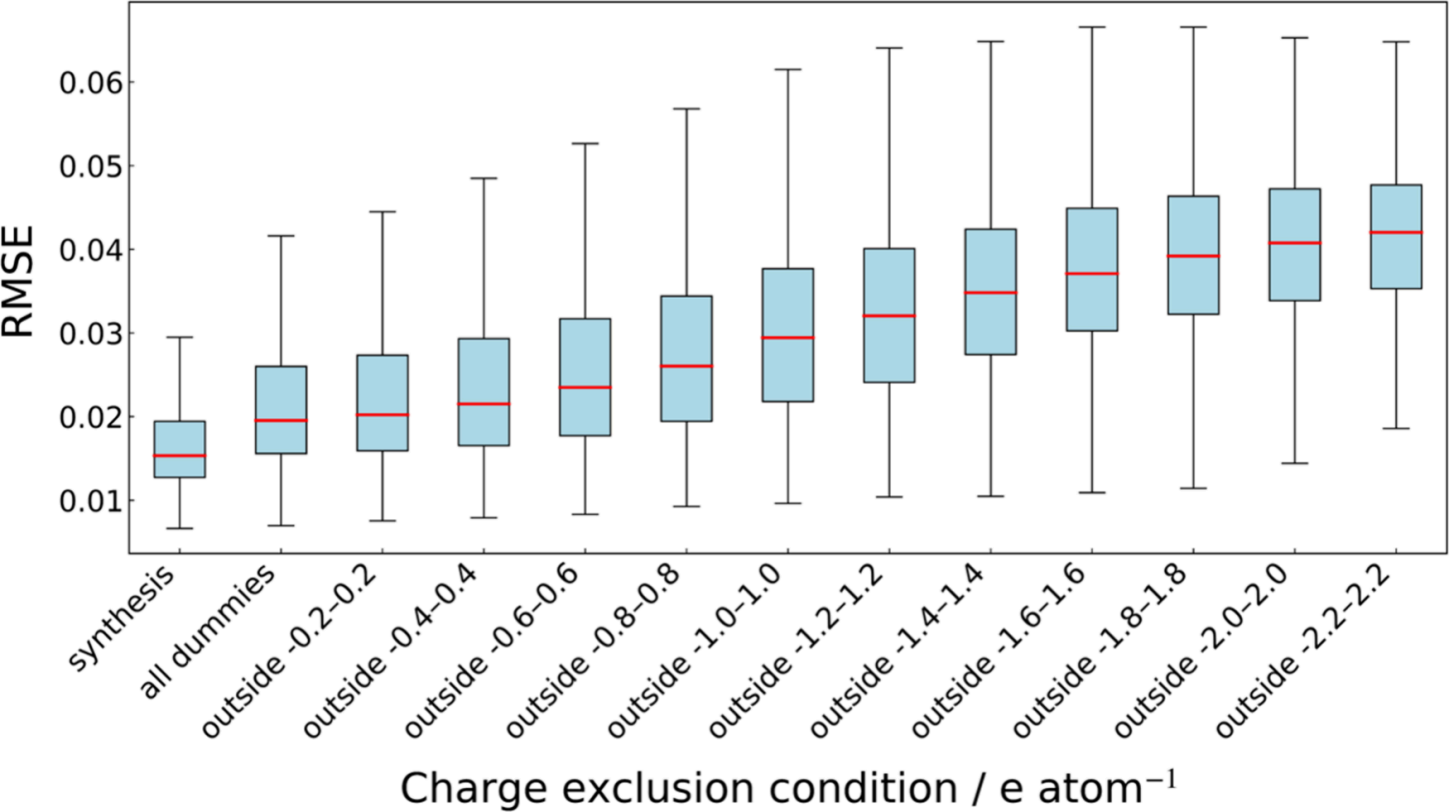
Changes in the RMSE distribution for 50,000 dummy oxide compositions with intentionally perturbed charge balance. Compositions whose total charge (*Q*) satisfies – threshold < *Q* < + threshold excluded; by increasing the threshold, the deviation from charge neutrality is progressively enlarged.

As the threshold increased (i.e., as the compositions were farther from charge neutrality), the RMSE distribution shifted toward higher values, clearly indicating that the greater the total charge imbalance, the more likely the model was to consider a composition to be anomalous. This behavior suggests that because the model was trained on many positive examples of compositions that satisfied charge neutrality, it tended to estimate a lower RMSE for charge-neutral compositions, even without explicitly specifying the typical oxidation states of the elements, whereby deviations from charge neutrality could lead to destabilization of the material.

Moreover, we extracted 1254 oxides composed only of the above elements from the Materials Project that were used as training data (energy above hull <0.01 eV/atom), computed their formal charges using the same procedure as in the previous section, and evaluated their relationship with RMSE (Figure 4). In general, a larger deviation from charge neutrality corresponded to a higher RMSE. However, charge-neutral compositions with large RMSE were also observed, and compositions that deviated from charge neutrality but exhibited comparatively small RMSE values were identified. Accordingly, the analysis was first restricted to compositions satisfying charge neutrality (charge = 0), and the 50 lowest and 50 highest RMSE values were examined.

**Figure 4:**
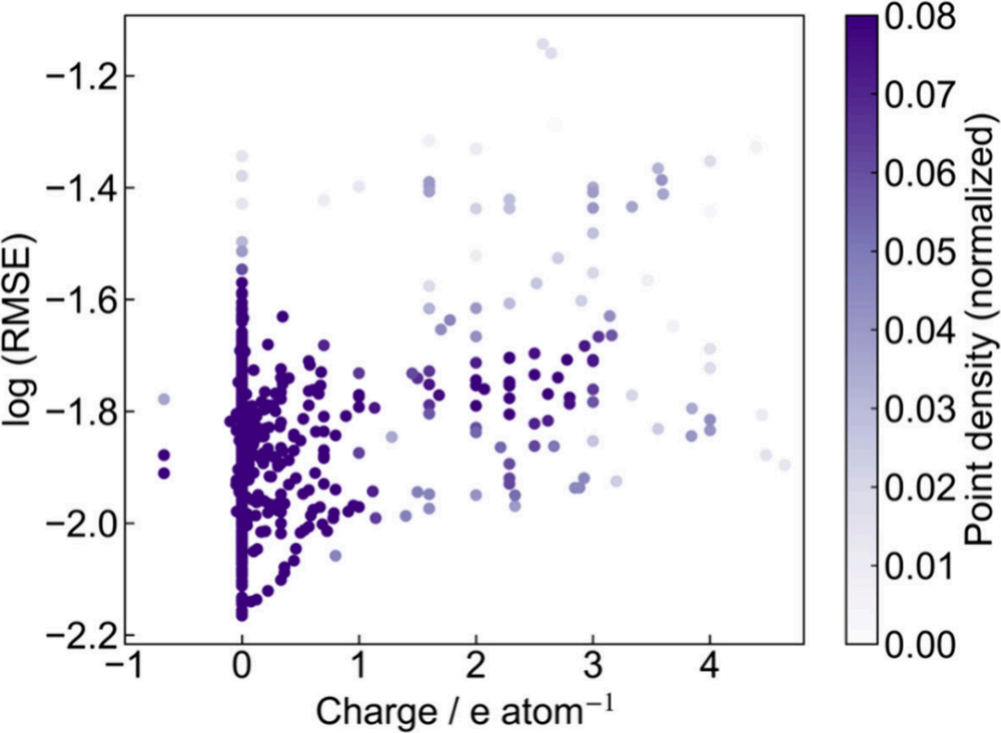
Relationship between the total formal charge per atom and the RMSE for oxides contained in the training data (Materials Project, energy above hull <0.01 eV/atom). The horizontal axis shows the total formal charge per atom, and the vertical axis shows the common logarithm of the autoencoder’s reconstruction RMSE. Formal charges were computed based on the representative oxidation states of each element (see main text). The color of each point represents the local point density estimated by Gaussian kernel density estimation (KDE), and the color bar shows the density values normalized to 0–0.05. Higher values (darker colors) indicate higher point densities.

In the low-RMSE group (Table S6 in the Supporting Information), ternaries were the most frequent, and main-group elements such as P and Si appeared at a high frequency. By contrast, in the high-RMSE group (Table S7 in the Supporting Information), quaternaries were the most frequent, with non-negligible numbers of compositions with five or six elements, and transition metals such as Hf and Ti appeared at a high frequency. These observations suggest that an increase in the number of element types indicated greater compositional complexity, and the presence of specific elements—such as transition metals—could contribute to larger RMSE values. Moreover, compositions that were clearly oxygen-deficient (for example, Al_4_Zn_2_Zr_6_O, Al_3_OZn_3_Zr_6_, and Al_7_O_2_Zn_5_Zr_12_) were identified as exhibiting comparatively small RMSE values despite not satisfying charge compensation. These are examples of suboxides; in the Materials Project, several suboxides are registered—such as B_6_O, Zr_4_O, Ti_8_BiO_7_, and Zr_10_Al_6_O—making it likely that the model learned the characteristic features of such compounds. Notably, experimentally reported syntheses exist for oxygen-deficient compositions. For example, Ti_8_BiO_7_ ^43^ has been synthesized and structurally characterized. Therefore, a low RMSE value could reasonably occur for apparently charge-imbalanced compositions when their compositional patterns resembled those of known realizable suboxide-type compounds. Moreover, as a different type of case exhibiting a small RMSE despite deviating from charge neutrality, compositions such as La_2_S_2_O and Ba_3_OSb_4_ could be identified in which S and Sb behaved as 2^–^ and 3^–^ anions rather than as 6^+^ and 5^+^ cations, as assumed in the present setting. Thus, even when elements capable of multiple valence states, such as S and Sb, were included, the RMSE could decrease if the compositional arrangement was similar to the learned features. These results indicate that the model did not rely solely on the magnitude of energy above hull or on the (formal) charge, but also learned other compositional features present in the descriptor space and used them to assess the synthesizability.

We then searched for quaternary chemical formulas in accessible peer-reviewed papers published in 2025, extracting 49 compositions that were not listed in the Materials Project for which experimental synthesis was confirmed from the text, and evaluated their RMSE values (Table S8 and Figure S3 in the Supporting Information). Approximately half of the materials exhibited relatively low RMSE values (RMSE < 0.02), and 45 materials exhibited RMSE values <0.03. As only a limited number of compositions exhibited extremely large RMSE values, these results suggest that the autoencoder-based anomaly detection was effective.

The results of analyzing the contribution to the anomaly scores (reconstruction error) using Kernel SHAP for the model are shown in Figure 5. Among the top 20 features, many were spdf-type descriptors, period numbers (PNs), and group numbers (PGs). In particular, a large proportion of features represented elemental combinations, such as the SPDF matrix (descriptors derived from matrices that combined the spdf descriptors of two elements in a composition), spdf–spdf (differences between two spdf descriptors), and spdfspdf (products).

**Figure 5:**
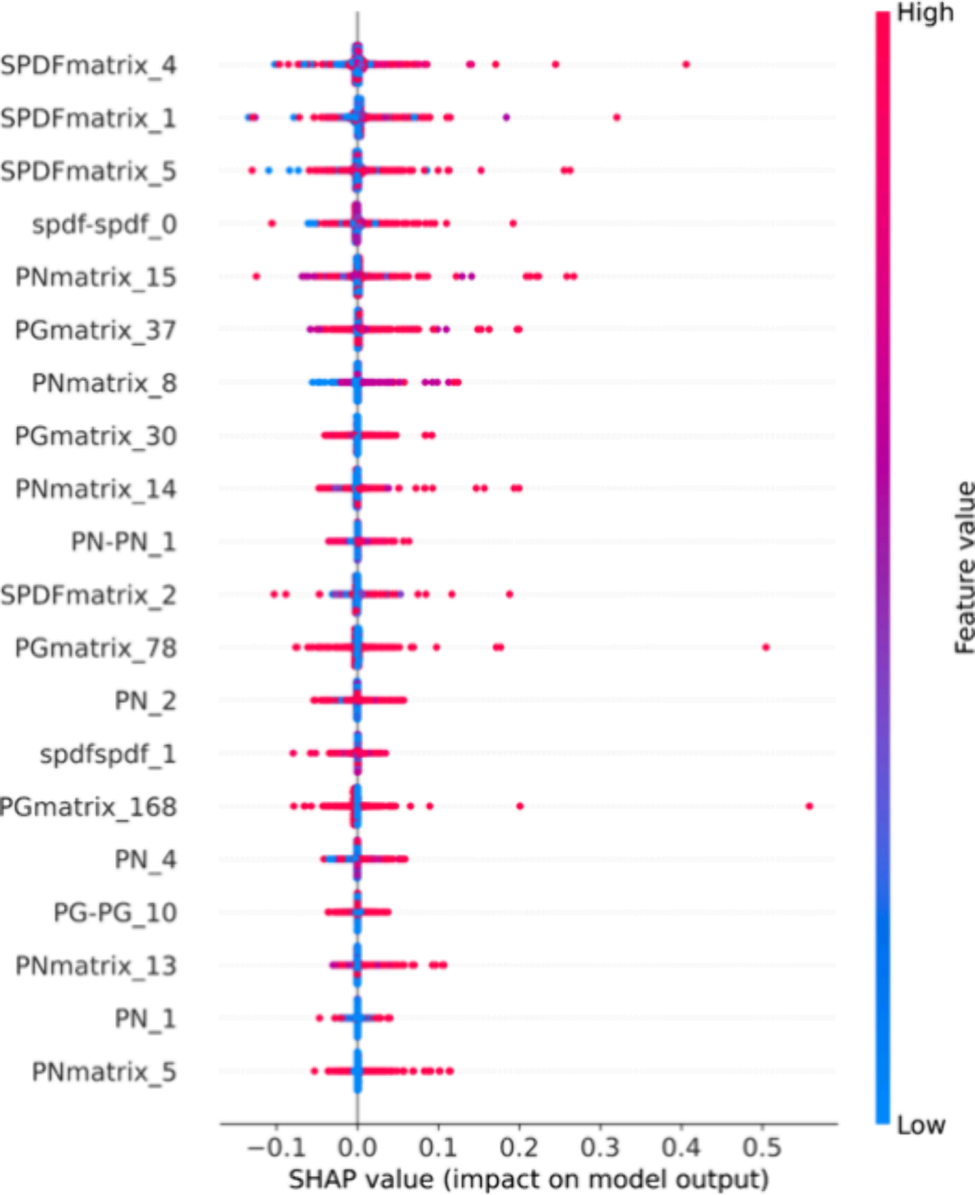
Contribution distribution of the input features by Kernel SHAP. The horizontal axis shows the SHAP values (influence on the reconstruction output), and the point colors indicate the magnitude of each feature value, from low to high. The vertical spread of points reflects regions with high point density. Among the evaluation data, the top 10 samples with the largest reconstruction errors were selected; for each sample, Kernel SHAP was applied to the decoder outputs that collectively accounted for the majority of the error (e.g., 80%). The resulting SHAP values were aggregated over samples × outputs, and the top 20 features were visualized.

The results suggest that rather than the properties of individual elements alone, elemental combinations (interactions) strongly influenced the determination of anomaly scores. In other words, when considering synthesizability from composition descriptors, the model was likely to identify combinations of elemental pairs that were easier or more difficult to realize. Moreover, the PG and spdf indirectly reflected trends in the valence states, supporting the detection of charge deviation, as shown in Figure 3. Additionally, the PN and PG encompassed periodic properties—such as ionic radius and electronegativity—and could be inferred to function effectively in capturing trends related to the structure and stability.

From importance analysis of the contribution of each descriptor to the anomaly score, it was suggested that elemental combinations (descriptors derived from two elemental properties) were the principal factors governing the reconstruction RMSE. Accordingly, from the element set used in Figures 3 and 4, we generated 50,000 virtual compositions constrained to include 2–4 cations and to satisfy charge compensation as oxides, and computed their RMSE. The distribution of the median RMSE for each combination is shown in Figure 6(a).

**Figure 6:**
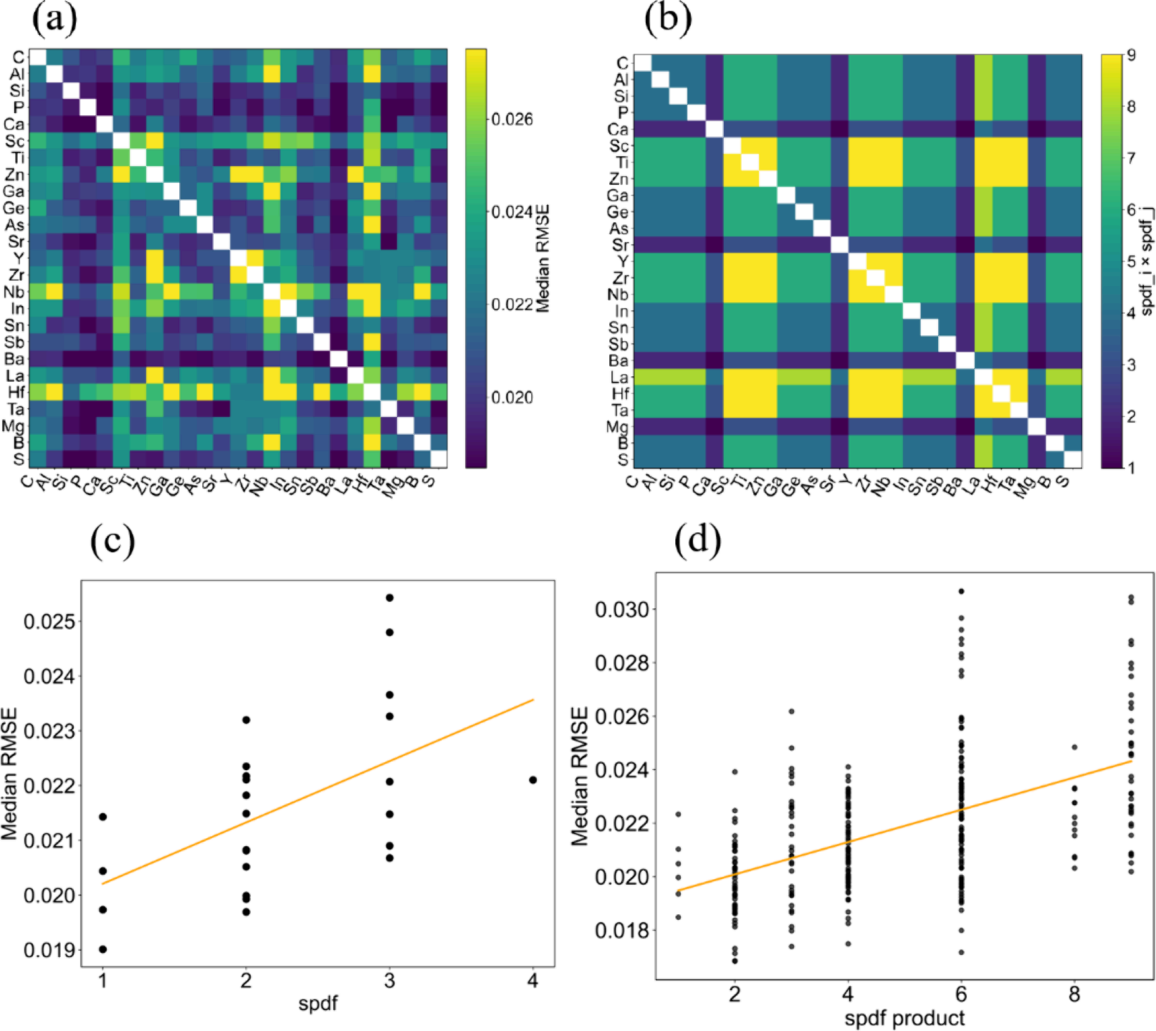
(a) Distribution of the reconstruction RMSE for cation combinations (heat map). The horizontal and vertical axes list the cation species; each cell summarizes the median reconstruction RMSE taken over all virtual oxide compositions that contain the corresponding cation pair (i.e., the median across all stoichiometries and any additional cations in which that pair appears). A total of 50,000 charge-compensated virtual oxides (each comprising 2–4 cations) were generated from the element set used in Figures 3 and 4, and the RMSE was computed for each composition. (b) Cations belonging to the s-, p-, d-, and f-blocks were assigned orbital indices of 1, 2, 3, and 4, respectively, and the products of these indices were calculated for each cation pair. The resulting values are shown as a heat map. (c) Relationship between the element-wise median reconstruction RMSE and valence-shell orbital type. (d) Relationship between the pairwise median reconstruction RMSE and spdf product for the cation pairs. The horizontal axis shows the spdf orbital index product for each cation pair. Orange lines in panels (c) and (d) correspond to least-squares linear regression lines.

Overall, compositions containing main-group elements tended to exhibit lower RMSE values, whereas those containing transition-metal elements tended to exhibit higher RMSE values. This was consistent with the trend observed for charge-neutral oxides, as shown in Figure 4. Because the feature-importance analysis described above indicates that many of the top-ranked features were constructed from combinations of spdf descriptors of the two elements, we inferred that the combinations of spdf types between element pairs contributed to this RMSE distribution. Consequently, we assigned values of 1, 2, 3, and 4 to the s-, p-, d-, and f-block elements (orbital index) and calculated the product of these spdf values for each element pair, the distribution of which is shown in Figure 6(b). An approximately monotonic relationship could be observed when this result was compared to the distribution of the median RMSE (Figure 6(a)).

To quantitatively examine the correspondence for each element, we first defined the median RMSE over all compositions containing that element as its representative value and plotted it against the type of valence-shell orbital (s, p, d, f) (Figure 6(c)). As an ordinal index, the median RMSE exhibited a monotonically increasing trend, although the variation in the RMSE values was large. Next, for all the element pairs in Figure 6(b), we used the median RMSE as the representative value and plotted it against the spdf product of each pair (Figure 6(d)). Evidently, the RMSE tended to increase monotonically with the spdf product, corroborating that elemental combinations derived from the spdf orbital index contributed to the RMSE distribution. Specifically, pairs with smaller spdf orbital index products (s-s, s-p, p-p, or main-group elements) tended to have lower RMSE values, whereas pairs with larger spdf products (such as d or f orbitals) tended to have higher RMSE values. This indicates that even when only compositional descriptors were used, differences in the valence-shell orbital combinations enabled a coarse discrimination between element combinations corresponding to readily synthesizable compositions and those corresponding to less synthesizable ones.

However, compositions containing Ta partially deviated from this monotonic trend. Notably, Ta-containing compositions were not overrepresented in the training data set (of the order of 3.67% of all training compositions), suggesting that the observed deviation was unlikely to be a simple artifact of elemental-frequency bias. Moreover, Ta is a cation with a d-orbital and is therefore expected to exhibit a high RMSE because it yields a large spdf product; however, it consistently exhibited relatively low RMSE values in combination with many other elements. For Ta-containing compositions, it was likely that not only spdf product descriptors contributed to the model reconstruction. Alternatively, the effects associated with combinations of more than three elements, which were not explicitly analyzed in this study, could also have played a role.

Thus, although feature-importance analysis can provide hints for predicting synthesizability patterns, the actual judgment is governed by complex, nonlinear relationships among multiple descriptors. Therefore, AE-based anomaly detection can be considered effective as it can capture multidimensional descriptor interactions.

In summary, we report an autoencoder-based anomaly detection model that used composition descriptors as input was developed and trained on inorganic compounds with low energy above hull, a metric of thermal stability. Using the reconstruction error (RMSE) as the anomaly score, the RMSE increased as the energy above hull of the evaluated composition increased, indicating that decreases in thermodynamic stability were reflected as higher anomalies. Moreover, when 50,000 virtual oxides with deliberately disrupted charge balances were evaluated, the RMSE increased with the magnitude of the total charge imbalance, suggesting that the model could capture the relationship in which departures from charge neutrality led to destabilization, even without explicit charge labels. Kernel SHAP analysis indicated that the PN, PG, and spdf-derived pair features predominantly governed anomaly judgments. Additionally, for the 50,000 charge-compensated virtual oxides, the distribution of the median RMSE for each element combination corresponded approximately monotonically to the distribution of a simple spdf product for the combinations, as indicated by the Kernel SHAP analysis. By contrast, the pairs containing Ta departed from this trend and exhibited a comparatively low RMSE, suggesting that descriptor information beyond spdf related ones also influenced the assessment of synthesizability. These results show that even with composition-only information, a coarse yet interpretable landscape of synthesizability over a multicomponent composition space could be obtained, enabling rapid prescreening beyond existing databases. Here, we used first-principles calculations that reflected the 0 K limit from a data-availability perspective. However, in future work, we will systematically collect positive examples of compositions that have been experimentally synthesized as single phases as we expect that it will be possible to develop an anomaly detection system that can provide practical synthesis predictions for experimental researchers. Furthermore, we also envision a two-stage workflow in which multiple plausible crystal structures can be generated for promising compositions, prioritized by a low RMSE, and subsequently validated using first-principles calculations.

## Methods

In this study, we regarded the autoencoder reconstruction error as an anomaly score and used it to assess synthesizability. The model was trained using the mean squared error (MSE) as the loss function (eq 1) and optimized the weights by minimizing them. For anomaly evaluation, we used the RMSE (eq 2); materials with a larger RMSE are considered to deviate more from the normal cluster and are therefore deemed difficult to synthesize.

1 $$Loss=\frac{1}{d}\overset{d}{\underset{i=1}{\sum }}{\left({Out}_{i}-{In}_{i}\right)}^{2}$$

2 $$RMSE=\sqrt{\frac{1}{d}\overset{d}{\underset{i=1}{\sum }}{\left({Out}_{i}-{In}_{i}\right)}^{2}}$$

In this study, we tried several fully connected autoencoder models, where the number of encoded variables ranged from 2 to 32, and optimized a 13-layer fully connected network that started from an input of 1994 units, passed through 1024 → 512 → 256 → 128 → 64 → 32 → 16 → 32 → 64 → 128 → 256 → 512 → 1024, and reconstructed to an output of 1994 units (mentioned later). A rectified linear unit (ReLU) function was adopted as the activation function. The hyperparameter optimization library Optuna was employed to optimize the batch size and learning rate.^44^ A search was conducted over 500 training epochs. Among the candidates whose validation losses ranked within the top 10 epochs, the condition that minimized the gap between the training and validation losses was selected as the final hyperparameter setting. The search ranges were batch size = 64–1024 and learning rate = 1 × 10^–4^–1 × 10^–3^. The optimal values were batch size = 240 and learning rate = 8.23 × 10^–4^. The Adam optimizer was used, and with random seed 42, the data were split—that is, 70% for training and 30% for testing. To assess whether optimization problems—such as vanishing gradients or dead ReLU behavior—affected the training, we monitored the gradient/weight-update statistics across epochs and the fraction of zero activations in the ReLU layers. As shown in Figure S1, these diagnostics indicate stable learning without dominant signs of gradient collapse or widespread dead ReLU units.

For the importance analysis, we employed Kernel SHAP within the SHAP (SHapley Additive exPlanations) framework.^45,46^ For each output dimension (*i*) of the autoencoder, we defined a function *f*_*i*_ (x) that returned the *i* ^th^ component of the reconstruction of input *x* and estimated the Shapley values of the input features using Kernel SHAP. For the background distribution (baseline), 200 instances were randomly sampled from the training data set and summarized into representative points via *k*-means (K = 80). As explanatory targets, the top ten samples with the largest reconstruction error (total squared error) over the entire data set were selected and for each sample we explained only those output dimensions that collectively accounted for 80% of the output-side error (coverage = 0.8). The sampling design for Kernel SHAP was determined based on the trade-off between the estimation accuracy and computational cost. We used the official SHAP implementation (Kernel Explainer)^47,48^ and performed inferences on a GPU.

We did not apply PCA or other prereduction steps prior to training as such transformations could mix histogram- and pair-derived features and substantially reduce the interpretability of the anomaly score. Instead, we retained the original descriptor axes and used Kernel SHAP to identify the descriptors that dominated the reconstruction errors. The results indicate that only a subset of periodic-table-derived and element-pair features contributed strongly, suggesting that the high-dimensional input mainly provided a redundant representation of elemental and pairwise interactions rather than arbitrary noise.

## Supplementary Material



## Data Availability

The code, optimized parameters for the autoencoder, and descriptors are available at the FigShare repository: https://doi.org/10.6084/m9.figshare.30575660. Data for this study are available from the corresponding author upon request.

## Notes

Descriptor definitions and construction procedures, additional model diagnostics and statistical evaluations, extended RMSE distribution analyses, and supporting tables listing representative compositions and screening results (PDF)
