# Supplementary material for: Anomaly-Detection-Driven Screening of Thermodynamic Stability from Composition Descriptors Alone
Source: J Phys Chem Lett. 2026 Feb 10;17(7):1937–46. doi: 10.1021/acs.jpclett.5c03772 (PMC12927008; doi:10.1021/acs.jpclett.5c03772)
Supplement: Supplementary file 1 [file jz5c03772_si_001.pdf]

Supporting Information

Anomaly-Detection-Driven Screening of Thermodynamic Stability from Composition Descriptors Alone

Keisuke Makino[a], Yudai Yamaguchi[a], Naoto Tanibata[a], Hayami Takeda[a], Ryo Kobayashi[b], Masayuki Karasuyama[c], Masanobu Nakayama[a] **

[a] Department of Advanced Ceramics, Nagoya Institute of Technology, Nagoya, Aichi 466-8555, Japan;

[b] Department of Physical Science and Engineering, Nagoya Institute of Technology, Nagoya, Aichi 466-8555, Japan;

[c] Department of Computer Science, Nagoya Institute of Technology, Nagoya, Aichi 466-8555, Japan;

***** Corresponding author: masanobu@nitech.ac.jp

Section S1. Compositional Descriptors

This supporting section describes the compositional descriptors used in this study, particularly the construction methods of the **algebraic descriptor** and **matrix descriptor**. The Python implementation used for descriptor conversion from the chemical composition formulas is available on GitHub[1], along with detailed documentation and usage examples.

S1.1 Standard Compositional Histogram Descriptor

The standard compositional descriptor represents elemental properties such as electronegativity and atomic radius as a one-dimensional histogram (distribution function). In this approach, the ***x*-axis** corresponds to the elemental property values, and the ***y*-axis** represents the **atomic fraction** of each element in the composition, normalized such that the total number of atoms is equal to one.

To ensure continuity and differentiability suitable for machine learning, **Gaussian broadening** was applied to the histogram, resulting in a smooth continuous vector representation. The detailed formulation of this histogram descriptor is described in our previous work[2,3]. The default parameters used for histogram construction (minimum, maximum, bin count, and Gaussian width) are listed in **Table S1**[1].

S1.2 Algebraic Descriptor

An **algebraic descriptor** represents the pairwise interactions between different elements within a chemical composition. For each unique element pair (A–B), the difference or product of a selected elemental property P(e.g., the atomic number, electronegativity, and atomic radius) can be calculated as follows:

$$\text{Difference: } \Delta P = | P_A - P_B | \text{ orProduct: } P_A \times P_B$$

The atomic fraction of each element (x_1, x_2) can be computed from the normalized composition, and the **pair concentration** can be defined as the sum of their atomic fractions, as follows:

$$x_{\text{pair}} = x_1 + x_2$$

A histogram can then be constructed with the elemental property difference (or product) on the *x*-axis and the pair concentration x_{pair} on the *y*-axis. Gaussian broadening can be applied to the distribution to obtain a continuous representation.

Example:

For the composition $\mathbf{Li_{0.5}Mn_{1.0}O_{2.0}}$, the pair concentration for the Li–O pair can be computed as follows:

$$x_1 = 1/3.5 = 0.1429,\ x_2 = 2/3.5 = 0.5714,\ x_1 + x_2 = 0.7143.$$

where x_1 and x_2 denote the Li and O fractions, respectively. These values contribute to the histogram weight of the difference in the properties of Li and O. As a result, we can yield algebraic descriptors for electronegativity (**EN**), **ΔEN** (difference), **EN^2** (product), and Pair concentration as below (**Table S2**).

S1.3 Matrix Descriptor

The **matrix descriptor** represents the pairwise relationships between elements based on **categorical elemental properties**, such as:

- **PN**: principal quantum number

- **PG**: group number in the periodic table
- **spdf**: valence block type (s = 1, p = 2, d = 3, f = 4)

For each property, a square matrix can be constructed, in which the **rows and columns correspond to the category indices** of the property. Each matrix element M_{ij} stores the **sum of atomic fractions** $(x_1 + x_2)$ for all element pairs belonging to categories i and j. Importantly, **diagonal elements are included**. The diagonal terms M_{ii} represent pairs of different elements that share the same property category—such as Ni–Mn in the d-block or O–F in the p-block.

After the matrix is constructed, the **upper-triangular part** (including the diagonal) can be flattened row-by-row into a one-dimensional vector, which is used as the **matrix descriptor**.

Note on sparsity. As the matrix descriptor aggregates element pairs into periodic-table-informed categories (PN/PG/spdf) rather than enumerating all specific element pairs in a one-hot manner, the resulting vector does not become extremely sparse, even for simple compositions. Zero entries contain information, indicating that no element pairs in the composition fall into the corresponding category pair.

Example:

For the composition $\mathbf{Li_{1.0}Ni_{0.5}Mn_{0.5}O_{2.0}}$, the spdf matrix descriptor can be constructed from element pairs according to their block indices (s = 1, p = 2, d = 3) and atomic fraction *x* as listed in **Table S3**.

Hence, we can obtain an atomic pair matrix using the spdf indices, as shown in **Table S4**. For example, the Li-O (O-Li) pair (atomic fraction sum is 0.25 + 0.50 = 0.75) contributes to the matrix elements of ($spdf_1$, $spdf_2$)

= (1,2) and (2,1).   The resulting upper-triangular matrix in **Table S4** can be converted into a single vector descriptor: [0.000, 0.750, 0.750, 0.000, 0.000, 1.250, 0.000, 0.250, 0.000, 0.000].

Table S1. Elemental properties used for chemhist descriptors, and settings for histogram (distribution function) representation: Min, Max, and Bin correspond to the minimum value, maximum value, and number of divisions (bins) for the elemental property distribution, respectively. Sigma represents the width of the Gaussian broadening applied to the histogram.

Elemental Property	Abbreviation	Min	Max	Bin	Sigma
Atomic Number	AN	1	103	102	0
Electronegativity	EN	-1	5	50	0.2
Melting Point	MP	-1000	5000	50	200
Period Number	PN	-2	10	20	0.4
Group Number	PG	-4	20	20	0.8
Mendeleev Number	MN	-24	120	102	4.8
Atomic Weight	AW	-80	400	50	16
Atomic Radius	AR	-0.2	3.2	50	0.128
Ionic Radius	IR	-0.2	3.2	50	0.128
Covalent Radius	CoR	-0.2	3.2	50	0.128
Crystal Radius	CrR	-0.2	3.2	50	0.128
s-, p-, d-, f-block elements	spdf	1	5	4	0

Table S2. Example of algebraic descriptors for the atom pairs.

**Atom Pair**	**EN₁**	**EN₂**	**ΔEN**	**EN²**	**Atomic Fraction**		$x_1 + x_2$
					x_1	x_2	
Li–Mn	0.98	1.55	0.57	1.52	0.143	0.286	0.429
Li–O	0.98	3.44	2.46	3.37	0.143	0.571	0.714
Mn–O	1.55	3.44	1.89	5.33	0.286	0.571	0.857

Table S3. spdf indices and atomic fractions for $Li_{1.0}Ni_{0.5}Mn_{0.5}O_{2.0}$.

**Element**	**spdf**	**Atomic fraction (*x*)**
Li	1	1.0/4.0 = 0.25
Ni	3	0.5/4.0 = 0.125
Mn	3	0.5/4.0 = 0.125
O	2	2.0/4.0 = 0.50

Table S4. Combination of the spdf × spdf matrix

**$spdf_1$¥$spdf_2$**	1	2	3	4
**1 (s)**	0.000	0.750	0.750	0.000
**2 (p)**	0.750	0.000	1.250	0.000
**3 (d)**	0.750	1.250	0.250	0.000
**4 (f)**	0.000	0.000	0.000	0.000

Table S5. Top-50 entries with the smallest RMSE within the Materials Project[4–6] subset with energy above hull (E_{hull}) > 0.01 eV/atom (N = 62247). Columns list the composition, Materials Project ID, energy above hull (eV/atom), reconstruction RMSE, and presence/absence of a reported synthesis. Synthesis labels were assigned by direct inspection of the Inorganic Crystal Structure Database (ICSD[7]). RMSE values were computed using the composition-only autoencoder described in the main text.

Composition	mp-ID	Energy above hull (eV/atom)	RMSE	Reported synthesis
$B_{10}Ni_{19}Y_3$[8]	mp-1199550	0.0104	0.00669	○
Ga_3Ni_2Y[9]	mp-1188481	0.0943	0.00686	○
$B_9Ni_{15}Y_2$[10]	mp-1204725	0.0169	0.00690	○
Sn_3Zr_4	mp-1094315	0.2539	0.00702	×
$Zr_2P_3O_{12}$	mp-774483	0.0136	0.00704	×
Nb_5S_8	mp-1220654	0.0106	0.00705	×
$Ni_{16}Si_7Y_6$	mp-1207874	0.0151	0.00707	×
SnZr	mp-1094260	0.1533	0.00709	×
ZrP_2O_8[11]	mp-1201586	0.1765	0.00710	○
Ni_5P_2[12]	mp-1200923	0.0168	0.00711	○
$Cu_{11}S_{16}$	mp-675278	0.2373	0.00714	×
B_4Rh_4Y[13]	mp-5984	0.0127	0.00715	○
$Ni_{25}Si_9$	mp-1209041	0.0116	0.00718	×
$Ni_{12}Sc_4Si_7$	mp-567443	0.0394	0.00720	×
Ga_4Zr_5[14]	mp-1188336	0.0466	0.00721	○
$NaZrP_2O_8$	mp-733652	0.0953	0.00728	×
$Ir_3Si_3Y_2$	mp-1206214	1.7931	0.00729	×
Cu_9S_{16}	mp-685056	0.577	0.00732	×
$Sn_2P_5O_{15}$	mp-26950	0.0364	0.00734	×
$Cu_{11}S_{20}$	mp-684964	0.281	0.00736	×
$Ca_6Si_3O_{13}$	mp-734368	0.0913	0.00738	×
$Ag_7P_3S_{11}$[15]	mp-683910	0.0192	0.00743	○
$Ca_6H_2Si_3O_{13}$[16]	mp-554539	0.0107	0.00745	○

$Ba_2Sb_2O_7$	mp-752404	0.0374	0.00745	×
Nb_7S_8	mp-1220475	0.0694	0.00745	×
$Ca_5Si_2O_{10}$[17]	mp-1197843	0.0706	0.00747	○
NiP[18]	mp-27844	0.0161	0.00749	○
Ca_3SiO_5[19]	mp-641754	0.0201	0.00751	○
P_2S_3[20]	mp-29014	0.0114	0.00753	○
$Zr_2P_4O_{17}$	mp-1215996	0.2604	0.00753	×
Zr_5Sb_4[21]	mp-570196	0.0246	0.00755	○
B_2Ru_2Y	mp-1208918	0.5929	0.00756	×
$Ni_6Sm_3Sn_4$	mp-1209076	0.1338	0.00756	×
$KNbSe_2$	mp-7940	0.0290	0.00757	×
$Nb_{11}S_{12}$	mp-684971	0.1744	0.00759	×
Al_8ErNi_3	mp-1225606	0.2709	0.00760	×
$PbPO_4$[22]	mp-1102025	0.1155	0.00765	○
$NaZrP_2O_9$[23]	mp-1180130	0.4485	0.00766	○
$CsCu_4S_3$[24]	mp-7785	0.0536	0.00766	○
$Pb_2P_3O_{10}$	mp-729974	0.0386	0.00769	×
$Ca_4Si_3O_{11}$[25]	mp-1202749	0.1211	0.00770	○
$Zn_5H_{18}P_6O_{30}$[26]	mp-1197483	0.0418	0.00771	○
$Sn_2P_3O_{10}$	mp-26944	0.0785	0.00776	×
$RbSO_4$[27]	mp-1190618	0.0669	0.00778	○
SnP_2O_9	mp-729138	0.2804	0.00778	×
$Ga_{17}Nd_4Ni_2$	mp-1220632	0.0946	0.00781	×
H_4SeZnO_6[28]	mp-605344	0.0176	0.00781	○
Na_2SO_5[29]	mp-1180310	0.9473	0.00782	○
H_3ZnPO_5[30]	mp-707311	0.0121	0.00783	○
Sb_4O_9	mp-1041270	0.2258	0.00783	×

Table S6. Top-50 lowest-RMSE cases among charge-neutral (Charge = 0) oxides from the same 1254-entry training subset (E_{hull} < 0.01 eV/atom). Columns list the composition, Materials Project ID, energy above hull (eV/atom), and reconstruction RMSE. The RMSE values were obtained with the composition-only autoencoder; charge neutrality was determined as in the preceding analysis.

**Composition**	**mp-ID**	**Energy above hull (eV/atom)**	**RMSE**
ZrP_2O_7	mp-5024	0.0000	0.00684
ZrP_4O_{12}	mp-557909	0.0000	0.00691
$Ba_4Sb_2O_9$	mp-801028	0.0000	0.00699
SnP_4O_{12}	mp-25908	0.0090	0.00717
Ca_2SiO_4	mp-4481	0.0000	0.00726
$Ca_8Si_5O_{18}$	mp-1019570	0.0006	0.00728
$Ca_3Si_2O_7$	mp-585516	0.0024	0.00732
SnP_2O_7	mp-757192	0.0000	0.00737
ZnP_2O_6	mp-8230	0.0000	0.00773
$CaSiO_3$	mp-4429	0.0000	0.00775
$Zr_2P_2O_9$	mp-27132	0.0000	0.00777
$Zn_2P_2O_7$	mp-559319	0.0000	0.00786
Sb_2O_5	mp-1705	0.0000	0.00790
$BaZrP_2O_8$	mp-556139	0.0000	0.00806
$BaSb_2O_6$	mp-9127	0.0000	0.00816
ZnP_4O_{11}	mp-15438	0.0000	0.00817
$BaSiO_3$	mp-776084	0.0000	0.00825
SnO_2	mp-856	0.0000	0.00825
$LaSbO_4$	mp-1190427	0.0000	0.00830
Ba_2SiO_4	mp-17612	0.0000	0.00845
$Zn_3P_2O_8$	mp-557920	0.0000	0.00848
$Ba_2Si_3O_8$	mp-29222	0.0000	0.00853
$Ba_5Si_8O_{21}$	mp-669333	0.0050	0.00861
$Ba_3Si_5O_{13}$	mp-29223	0.0026	0.00866

$CaZr_4P_6O_{24}$	mp-556440	0.0000	0.00869
$CaZrP_2O_8$	mp-554495	0.0000	0.00870
$Sr_2Ta_2O_7$	mp-13664	0.0000	0.00876
$Zn_2As_2O_7$	mp-779594	0.0000	0.00877
$Sr_5Ta_4O_{15}$	mp-769582	0.0000	0.00878
$BaSi_2O_5$	mp-3031	0.0000	0.00888
$SbPO_5$	mp-9750	0.0000	0.00892
Ba_3SiO_5	mp-7765	0.0041	0.00894
La_3SbO_7	mp-1211549	0.0000	0.00900
Ta_2O_5	mp-10390	0.0000	0.00904
$BaZnP_2O_7$	mp-14345	0.0000	0.00908
$Y_2Si_2O_7$	mp-581644	0.0000	0.00918
$Ca_2ZrSi_4O_{12}$	mp-560339	0.0062	0.00919
$Ba_3CaSb_2O_9$	mp-554817	0.0000	0.00923
$Ca_2Sb_2O_7$	mp-16280	0.0000	0.00924
$LaSb_3O_9$	mp-31418	0.0000	0.00925
$BaZr_4P_6O_{24}$	mp-560308	0.0000	0.00927
$Sr_4Ta_2O_9$	mp-2205489	0.0000	0.00927
$As_2Zn_3O_8$	mp-16834	0.0000	0.00927
$Ba_4Sn_3O_{10}$	mp-2755271	0.0000	0.00930
$SrZnP_2O_7$	mp-558616	0.0000	0.00931
$Ba_3Sn_2O_7$	mp-2205007	0.0000	0.00934
$Ba_9Y_2Si_6O_{24}$	mp-1019533	0.0054	0.00936
YP_5O_{14}	mp-556335	0.0000	0.00938
$Ba_3YB_3O_9$	mp-6310	0.0000	0.00938
$BaSnO_3$	mp-3163	0.0000	0.00939

Table S7. Top-50 highest-RMSE cases among charge-neutral (Charge = 0) oxides drawn from the 1254-entry Materials Project training subset (E_{hull} < 0.01 eV/atom; elements as defined in the main text). Columns list the composition, Materials Project ID, energy above hull (eV/atom), and reconstruction RMSE. Charge neutrality was assessed using representative oxidation states (see Methods/previous section).

**Composition**	**mp-ID**	**Energy above hull (eV/atom)**	**RMSE**
$BNbO_4$	mp-8615	0.0000	0.04532
$B_3Mg_5NbO_{12}$	mp-1196847	0.0000	0.04176
$AsBBaO_5$	mp-9784	0.0000	0.03725
B_2BaHfO_6	mp-1105495	0.0000	0.03185
$As_6Hf_4SrO_{24}$	mp-3212620	0.0000	0.03062
$As_3Hf_2PO_{14}$	mp-3209819	0.0000	0.02844
In_2NbSbO_8	mp-3215473	0.0000	0.02692
$Ca_3Ga_3SbSi_2O_{14}$	mp-3213029	0.0000	0.02584
$As_6CaHf_4O_{24}$	mp-3206796	0.0000	0.02565
$Hf_2Y_4ZrO_{12}$	mp-3210844	0.0000	0.02479
$BaSrY_4O_8$	mp-1227529	0.0056	0.02428
B_2HfO_5	mp-559617	0.0000	0.02409
$ScSr_2SbO_6$	mp-1106218	0.0000	0.02401
$BaGaScZn_3O_7$	mp-1192501	0.0062	0.02359
Ga_4TiO_8	mp-553991	0.0041	0.02337
$MgZn_7O_8$	mp-1210610	0.0062	0.02336
HfP_2STiO_{12}	mp-2713475	0.0011	0.02335
$Al_9LaTi_2O_{19}$	mp-1197399	0.0018	0.02313
$Y_4Zr_3O_{12}$	mp-1216160	0.0000	0.02311
$Ga_5La_3TiO_{14}$	mp-1211258	0.0000	0.02306
As_4HfTiO_{14}	mp-3207990	0.0000	0.02291
$ZnCO_3$	mp-9812	0.0000	0.02197
$Ga_5HfLa_3O_{14}$	mp-1211281	0.0000	0.02182
HfO_2	mp-352	0.0000	0.02171
$B_{10}Ca_5Sn_4TiO_{30}$	mp-1227225	0.0052	0.02170

$HfSr_2O_4$	mp-754761	0.0064	0.02159
$SrCO_3$	mp-3822	0.0000	0.02158
$Al_2Ba_5Y_2ZrO_{13}$	mp-1214494	0.0085	0.02151
$Ge_2MgSr_2O_7$	mp-972387	0.0000	0.02142
Sc_2TiO_5	mp-753401	0.0000	0.02140
$Ba_3SbScTiO_9$	mp-3214492	0.0000	0.02139
$Ba_3In_2Zn_5O_{11}$	mp-560544	0.0000	0.02138
$As_6GaNb_3O_{24}$	mp-3208754	0.0000	0.02124
$Ca_3Ga_3NbSi_2O_{14}$	mp-1214038	0.0054	0.02112
$Al_3B_8Ga_3Y_2O_{24}$	mp-1216259	0.0051	0.02111
C_2CaZnO_6	mp-1078391	0.0013	0.02086
$B_3Mg_5TaO_{12}$	mp-1196921	0.0000	0.02074
$GeNbP_3O_{12}$	mp-2713887	0.0010	0.02067
Sc_2SrO_4	mp-770771	0.0000	0.02057
$Hf_2Sr_3O_7$	mp-752691	0.0084	0.02053
In_2SrO_4	mp-540688	0.0000	0.02050
B_2BaTiO_6	mp-11659	0.0028	0.02048
$SbTiP_3O_{12}$	mp-1043703	0.0000	0.02048
$YZr_6P_9O_{36}$	mp-1208165	0.0079	0.02041
$CaSrC_2O_6$	mp-559653	0.0091	0.02041
$Sc_2Ti_2O_7$	mp-753802	0.0000	0.02034
$Sr_{12}Ta_3SbO_{22}$	mp-3209059	0.0000	0.02022
$Ba_2Ge_2MgO_7$	mp-1190545	0.0000	0.02021
$Al_8SrTi_3O_{19}$	mp-1217828	0.0000	0.02010
$AlAs_6Nb_3O_{24}$	mp-3212090	0.0000	0.01998

Table S8. List of the reported inorganic compounds used for the out-of-database screening test. Compositions were collected from the literature published in 2025 or later and filtered to satisfy the following criteria: no overlap with compositions registered in the Materials Project, thermodynamically synthesized primary phases (excluding by-products/impurities), and inorganic compounds only. The table reports the composition of each material and reconstruction RMSE evaluated by the composition-only autoencoder described in the main text, providing an external test dataset for assessing screening behavior on compositions beyond the Materials Project training distribution.

Composition	RMSE
$Li_{1.6}Mn_{1.6}O_4$[31]	0.010074
Li_3VCl_6[32]	0.011624
$Na_2Fe_3(SO_4)_4$[33]	0.011959
Co_4S_3[34]	0.012176
$Ti_3C_2S_2$[35]	0.013346
$NaZrS_2$[36]	0.014147
Li_2TiS_3[37]	0.014545
$Ti_{0.96}Nb_{0.04}O_2$[38]	0.014634
$Li_{1.2}Ni_{0.24}Mn_{0.56}O_2$[39]	0.014770
Na_2MnO_2F[40]	0.015256
$Cs_3PMo_{12}O_{40}$[41]	0.016619
Li_2TaS_3[42]	0.017396
$Li_{1.2}Mn_{0.6}Nb_{0.2}O_2$[43]	0.017411
$Co_{0.5}P_{0.5}S_3$[44]	0.017518
$Li_{1.03}Mo_{0.02}Ni_{0.95}O_2$[45]	0.017817
$Li_{1.3}FeCl_4$[32]	0.018206
$Na_3CoB_5O_{10}$[46]	0.018362
$Li_{1.1}Ni_{0.875}S_{0.025}O_2$[45]	0.018498
$Li_{1.3}Fe_{1.2}Cl_4$[32]	0.018536
$Li_{1.15}Mn_{0.55}Ti_{0.3}O_2$[43]	0.019272
Pb_2CoRuO_7[47]	0.019712
$Li_{1.1}Mn_{0.7}Ti_{0.2}O_2$[43]	0.019990
$Li_{1.75}FeCl_4$[48]	0.020403
$Li_{1.05}Mn_{0.85}Ti_{0.1}O_2$[43]	0.020919
Li_3TiCl_6[32]	0.021083
$Li_3LaP_2S_8$[49]	0.023009
$Li_{1.06}Ni_{0.937}Zr_{0.04}O_2$[45]	0.023047

$Mn_{0.2}RuO_2$[50]	0.023511
$LiNi_{0.98}W_{0.02}O_2$[45]	0.024392
$LiNi_{0.985}W_{0.015}O_2$[45]	0.024484
$LiNi_{0.99}W_{0.01}O_2$[45]	0.024576
$Ni_{0.5}P_{0.5}S_3$[44]	0.024626
$K_2V_2Ti_6O_{16}$[51]	0.026578
$K_{1.75}V_{1.75}Ti_{6.25}O_{16}$[51]	0.026859
$LiMn_{0.5}Ti_{0.5}S_2$[37]	0.026894
$LaCo_{0.8}Ni_{0.2}O_3$[52]	0.027127
$K_{1.5}V_{1.5}Ti_{6.5}O_{16}$[51]	0.027231
$K_{1.5}V_{1.25}Ti_{6.75}O_{16}$[51]	0.027348
$Li_{2.1}Ga_{0.1}Zr_{0.9}Cl_6$[53]	0.027419
$LaCo_{0.85}Ni_{0.15}O_3$[52]	0.027423
$K_{1.5}VTi_7O_{16}$[51]	0.027503
$LaCo_{0.9}Ni_{0.1}O_3$[52]	0.027721
$K_{1.5}V_{0.75}Ti_{7.25}O_{16}$[51]	0.027732
$K_{1.5}V_{0.5}Ti_{7.5}O_{16}$[51]	0.028024
$K_{1.5}V_{0.25}Ti_{7.75}O_{16}$[51]	0.028379
$Li_{2.9}Fe_{0.9}Zr_{0.1}Cl_6$[32]	0.035073
$LiNi_{0.995}Nb_{0.005}O_2$[45]	0.040161
$LiNi_{0.99}Nb_{0.01}O_2$[45]	0.040193
$LiNi_{0.98}Nb_{0.02}O_2$[45]	0.040259

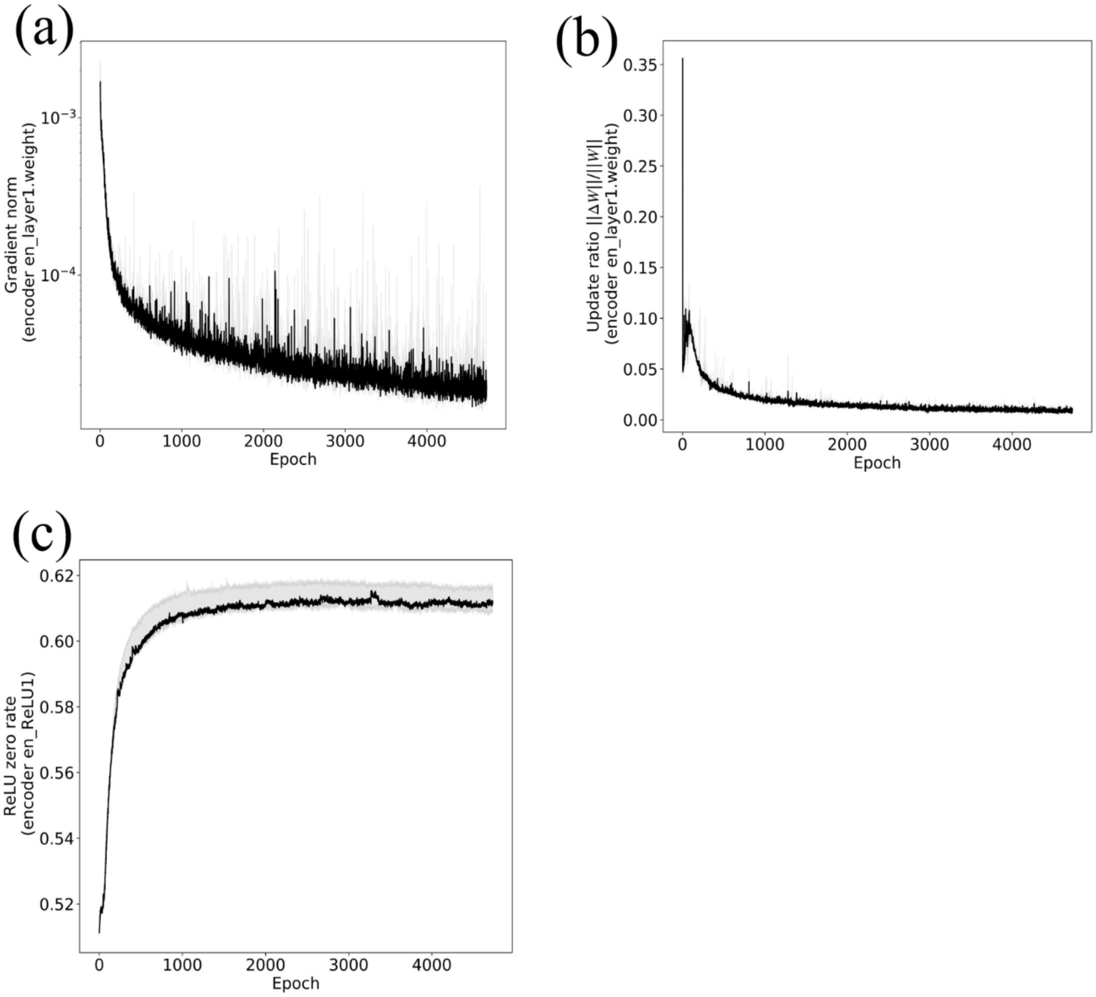

Figure S1. Training diagnostics of the autoencoder. (a) Gradient norm of the first encoder-layer weights during training. (b) Weight-update ratio, $||\Delta W||/||W||$, for the same layer. (c) Zero-activation rate of the first ReLU layer (fraction of activations equal to 0). In all panels, the black curve shows the median across the five folds of K-fold cross-validation, and the light-gray band indicates the interquartile range (IQR).

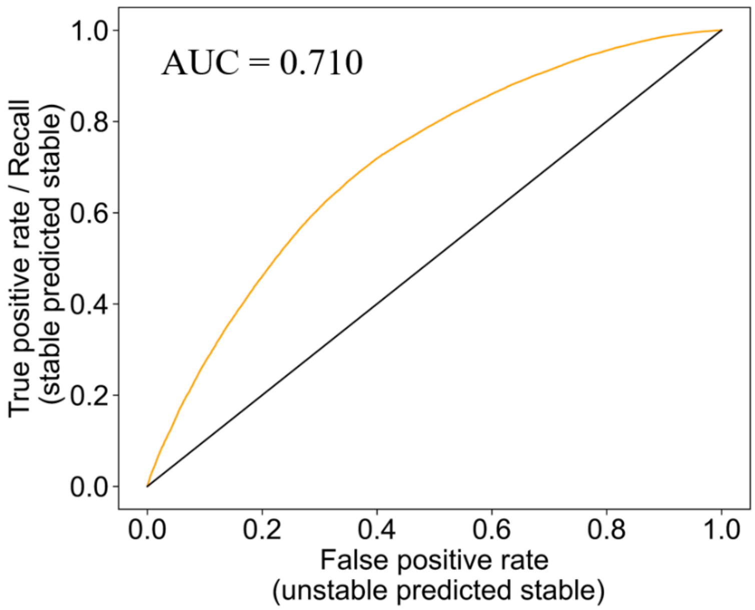

Figure S2. Receiver operating characteristic (ROC) analysis of the RMSE-based screening on the Materials Project dataset. Stable compounds were defined as $E_{hull} < 0.01$ eV/atom (positive class; the region used for training), and unstable compounds as $E_{hull} > 0.01$ eV/atom (negative class), both taken from the Materials Project. The ROC curve (orange) was generated by sweeping the decision threshold on the reconstruction RMSE. The diagonal line (black) represents random classification ($y = x$).

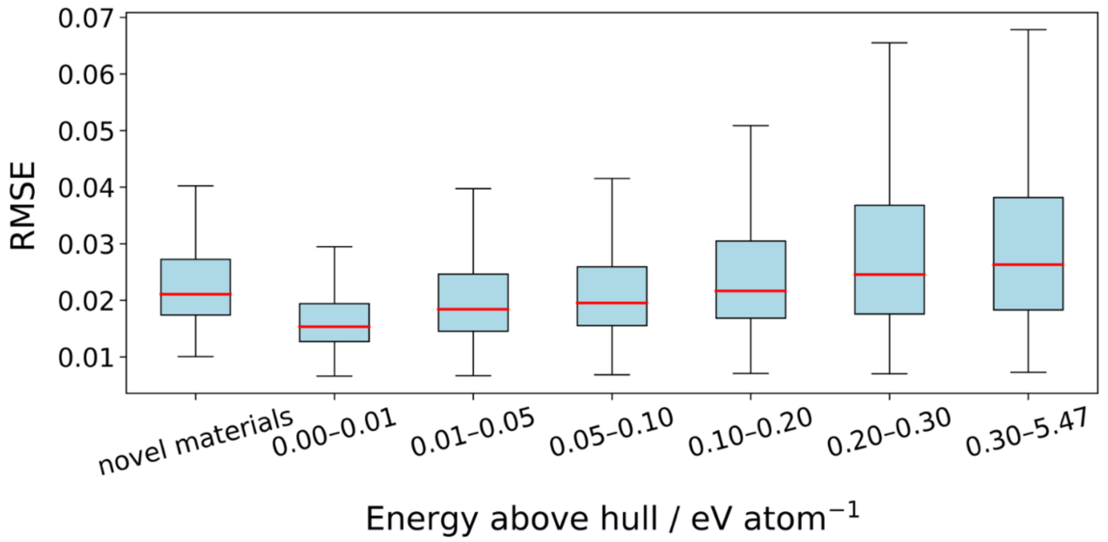

Figure S3. RMSE distributions across the thermodynamic-stability bins in the Materials Project, with additional materials listed in **Table S8** appended to **Figure 2**. Materials Project entries were grouped by energy above hull (E_{hull}; x-axis), where 0.00–0.01 eV/atom corresponds to the training region, and the reconstruction RMSE of the composition-only autoencoder was evaluated for each bin (y-axis). The leftmost box (novel materials) was created from the literature-derived compositions listed in **Table S8** and added to **Figure 2** to enable direct comparison with the E_{hull}-binned Materials Project distributions. Boxes indicate the interquartile range (IQR) with the median shown as a red line; whiskers follow the same convention as in **Figure 2**.

References

(1) Koyama, T.; Nakayama, M. *Compositional-Histogram-Descriptor*. GitHub. https://github.com/ChemicalBatteryLab-Nitech/compositional-histogram-descriptor (accessed 2025-11-07).

(2) Jalem, R.; Nakayama, M.; Noda, Y.; Le, T.; Takeuchi, I.; Tateyama, Y.; Yamazaki, H. A General Representation Scheme for Crystalline Solids Based on Voronoi-Tessellation Real Feature Values and Atomic Property Data. *Science & Technology of Advanced Materials* 2018, *19* (1), 231–242. https://doi.org/10.1080/14686996.2018.1439253.

(3) Yamaguchi, Y.; Atsumi, T.; Kanamori, K.; Tanibata, N.; Takeda, H.; Nakayama, M.; Karasuyama, M.; Takeuchi, I. Drawing a Materials Map with an Autoencoder for Lithium Ionic Conductors. *Scientific Reports* 2023, *13* (1), 16799. https://doi.org/10.1038/s41598-023-43921-1.

(4) Jain, A.; Hautier, G.; Ong, S. P.; Moore, C. J.; Fischer, C. C.; Persson, K. A.; Ceder, G. Formation Enthalpies by Mixing GGA and GGA \$+\$ \$U\$ Calculations. *Physical Review Part B* 2011, *84* (4), 45115. https://doi.org/10.1103/PhysRevB.84.045115.

(5) Jain, A.; Ong, S. P.; Hautier, G.; Chen, W.; Richards, W. D.; Dacek, S.; Cholia, S.; Gunter, D.; Skinner, D.; Ceder, G.; Persson, K. A. Commentary: The Materials Project: A Materials Genome Approach to Accelerating Materials Innovation. *APL Materials* 2013, *1* (1), 011002. https://doi.org/10.1063/1.4812323.

(6) Ong, S. P.; Richards, W. D.; Jain, A.; Hautier, G.; Kocher, M.; Cholia, S.; Gunter, D.; Chevrier, V. L.; Persson, K. A.; Ceder, G. Python Materials Genomics (Pymatgen): A Robust, Open-Source Python Library for Materials Analysis. *Computational Materials Science* 2013, *68*, 314–319. https://doi.org/https://doi.org/10.1016/j.commatsci.2012.10.028.

(7) Bergerhoff, G.; Hundt, R.; Sievers, R.; Brown, I. D. The Inorganic Crystal Structure Data Base. *Journal of Chemical Information & Computer Sciences* 1983, *23* (2), 66–69. https://doi.org/10.1021/ci00038a003.

(8) Gubich, I. B.; Kuz'ma, Y. B. New Borides of Rare-Earth Metals and Nickel with a Structure of the HO3NI19B10 TYPE. *Inorganic Materials* 1991, *27* (8), 1363–1365.

(9) Drasner, A.; Blazina, Z. Crystal Structure and Hydrogen Sorption Properties of the YNi5-XGaX Alloys. *Acta Chimica Slovenica* 2008, *55*, 869–873.

(10) Gubich, I. B.; Kuz'ma, Y. B.; Chaban, N. F. New Rare Earth Borides with the HO2NI15B9 Structure. *Inorganic Materials* 1991, *27* (3), 415–417.

(11) Norby, P. Synchrotron Powder Diffraction Using Imaging Plates: Crystal Structure Determination and Rietveld Refinement. *Journal of Applied Crystallography* 1997, *30* (1), 21–30. https://doi.org/10.1107/S0021889896009995.

(12) Chikhrii, S. I.; Kuz'ma, Y. B. Phase Equilibria in the Tb-{Cr, Fe, Co, Ni}-P Systems at 1070 K. *Russian Journal of Inorganic Chemistry* 1990, *35* (12), 1821–1823.

(13) Misra, S. K.; Postma, H. Low-Temperature Ordered States of R Rh 4 B 4 (R= Rare Earth) Due to Dipole-Dipole and Exchange Interactions. *Physcal Review Part B* 1984, *30* (3), 1285. https://doi.org/10.1103/PhysRevB.30.1285.

(14) Schubert, K.; Meissner, H. G.; Pötzschke, M.; Rossteutscher, W.; Stolz, E. Einige Strukturdaten Metallischer Phasen (7). *Naturwissenschaften* 1962, 49 (3), 57. https://doi.org/10.1007/BF00595382.

(15) Brinkmann, C.; Eckert, H.; Wilmer, D.; Vogel, M.; auf der Günne, J. S.; Hoffbauer, W.; Rau, F.; Pfitzner, A. Re-Entrant Phase Transition of the Crystalline Ion Conductor Ag7P3S11. *Solid State Sci* 2004, *6* (10), 1077–1088.

(16) Safronov, A. N.; Nevskii, N. N.; Ilyukhin, V. V.; Belov, N. V. The Refinement of the Crystal Structure of the Cement Phase γ-C6S3H. *Doklady Akademi Nauk SSSR* 1981, *256*, 1387–1389.

(17) Kuznetsova, T. P.; Nevsky, N. N.; Ilyukhin, V. V; Belov, N. V. Refinement of the Crystal-Structure OF Calcium Chondrodite CA5 [SIO4] 2 (OH) 2= CA (OH) 2.2 CA2SIO4. *Kristallografiya*. Mezhdunarodnaya Kniga 39 Dimitrova UL., 113095 MOSCOW, 1980, pp 159–160.

(18) Larsson, E. An X-ray Investigation of the Ni-P System and the Crystal Structures of NiP and NiP2. *Arkiv for Kemi* 1965, *23* (34), 335–365.

(19) Ángeles, G.; De Vera, R. N.; Cuberos, A. J. M.; Aranda, M. A. G. Crystal Structure of Low Magnesium-Content Alite: Application to Rietveld Quantitative Phase Analysis. *Cement & Concrete*

Research 2008, *38* (11), 1261–1269. https://doi.org/10.1016/j.cemconres.2008.06.005.

(20) Blachnik, R.; Peukert, U.; Czediwoda, A.; Engelen, B.; Boldt, K. Die Molekulare Zusammensetzung von Erstarrten Phosphor-Schwefel-Schmelzen Und Die Kristallstruktur von B-P4S6. Zeitschrift für Anorganische & Allgemeine Chemie 1995, *621* (10), 1637–1643. https://doi.org/10.1002/zaac.19956211004.

(21) Romaka, V. V; Romaka, L.; Rogl, P.; Stadnyk, Y.; Melnychenko, N.; Korzh, R.; Duriagina, Z.; Horyn, A. Peculiarities of Thermoelectric Half-Heusler Phase Formation in Zr–Co–Sb Ternary System. *Journal of Alloys & Compounds* 2014, *585*, 448–454. https://doi.org/10.1016/j.jallcom.2013.09.097.

(22) Katrusiak, A.; Nelmes, R. J. Pressure Dependence of the Crystal Structure of Lead Hydrogen Phosphate (PbHPO4). *Journal of Physics: Condensed Matter* 1989, *1* (50), 10165. https://doi.org/10.1088/0953-8984/1/50/017.

(23) Rudolf, P. R.; Clearfield, A. X-Ray Powder Structure and Rietveld Refinement of the Monosodium Exchanged Monohydrate of Alpha.-Zirconium Phosphate, Zr (NaPO4)(HPO4). Cntdot. H2O. *Inorgic Chemistry* 1989, *28* (9), 1706–1710.

(24) Ghosh, B. P.; Chaudhury, M.; Nag, K. Electron Transport and Magnetic Properties of Some Mixed-Valent Alkalithiocuprates. *Journal of Solid-State Chemistry* 1983, *47* (3), 307–313. DOI: https://doi.org/10.1016/0022-4596(83)90023-3.

(25) Gard, J. A.; Taylor, H. F. W. The Crystal Structure of Foshagite. *Acta Crystallographica* 1960, *13* (10), 785–793. https://doi.org/10.1107/S0365110X60001898.

(26) Herschke, L.; Enkelmann, V.; Lieberwirth, I.; Wegner, G. The Role of Hydrogen Bonding in the Crystal Structures of Zinc Phosphate Hydrates. *Chemistry–A European Journal* 2004, *10* (11), 2795–2803. https://doi.org/10.1002/chem.200305693.

(27) Asahi, T.; Hasebe, K. Crystal Structure of the High-Pressure Phase of $RbHSO_4$. *Journal of the Physical Society of Japan* 1996, *65* (10), 3233–3236. https://doi.org/10.1143/JPSJ.65.3233.

(28) Krivovichev, S. V. Crystal Chemistry of Selenates with Mineral-like Structures. IV. Crystal Structure of $Zn(SeO_4)(H_2O)2$, a New Compound with a Mixed Framework of the Variscite Type. *Geology of Ore Deposits* 2007, *49* (7), 542–546. https://doi.org/10.1134/S1075701507070094.

(29) Die Kristallstruktur von Na2S · $5H_2O$, Na_2Se · $5H_2O$ Und Na_2Te · $5H_2O$. *Zeitschrift für Kristallographie - Crystalline Materials* 1965, *121* (2–4), 131–144. https://doi.org/doi:10.1524/zkri.1965.121.2-4.131.

(30) Riou, A.; Cudennec, Y.; Gerault, Y. Hydrogénophosphate de Zinc Hydrate. *Acta Crystallographica Section C Crystal Structure Communications* 1987, *43* (2), 194–197. https://doi.org/10.1107/S010827018709646X.

(31) Herrmann, L.; Bohn, N.; Pfau, A.; Kölbel, T.; Ehrenberg, H.; Binder, J. R.; Jeschull, F. Synthesis of Lithium Manganese Oxide and Ti-Substituted LMO Sorbents for Lithium Extraction in a

Spray-Drying Process. *ChemSusChem* 2025, *18* (13), e202500530. https://doi.org/https://doi.org/10.1002/cssc.202500530.

(32) Sun, G.; Song, Z.; Dai, Y.; Xiao, Z.; Zheng, X.; Luo, W. Redox-Active Halide Catholytes for Solid-State Lithium Batteries. *Advanced Science* 2025, *12* (47), e14215. https://doi.org/https://doi.org/10.1002/advs.202514215.

(33) Grebenshchikova, A.; Olchowka, J.; Simonin, L.; Yaroslavtsev, S.; Duttine, M.; Fauth, F.; Stievano, L.; Masquelier, C.; Croguennec, L. Na2Fe3(SO4)4: A Zero-Strain Sustainable Positive Electrode Material for Na-Ion Batteries. *Angewandte Chemie International Edition* 2025, *64* (44), e202511285. https://doi.org/https://doi.org/10.1002/anie.202511285.

(34) Koo, B.; Kim, W.; Kim, Y.; Cao, X.; Jang, J.; Ko, M. J. Stoichiometry-Controlled Cobalt Sulfide-Based Hole Transport Layers for Perovskite Solar Cells. *Small* 2025, *21* (48), e05726. https://doi.org/https://doi.org/10.1002/smll.202505726.

(35) Lam, D. Van; Nguyen, V. H.; Yoo, H.; Dung, D. T.; Syed, S. A.; Ha, J.; Oh, W.; Lee, S.-M.; Oh, I.-K. Dry Synthesis of Sulfur-Terminated MXene as Multifunctional Catalyst for Stable Lithium–Sulfur Batteries. *Small* 2025, *21* (22), e2411668. https://doi.org/https://doi.org/10.1002/smll.202411668.

(36) Liers, L.; Mereacre, L.; Li, H.; Mickenbecker, J.; Knapp, M.; Indris, S.; Behrens, M.; Mangelsen, S. Electrochemical Intercalation Reaction of Sodium into the Layered Transition Metal Dichalcogenide ZrS2─Influence of the Electrolyte Solvent. *Inorganic Chemistry* 2025, *64* (36), 18045–

18061. https://doi.org/10.1021/acs.inorgchem.5c02331.

(37) Shinoda, M.; Matsunoshita, K.; Nakayama, M.; Hiroi, S.; Ohara, K.; Abe, M.; Ishiguro, N.; Takahashi, Y.; Hasegawa, G.; Kuwata, N.; Iwama, T.; Masuda, T.; Suzuki, K.; Ishii, H.; Shao, Y.-C.; Shibata, D.; Irizawa, A.; Ohta, T.; Konuma, I.; Ohno, T.; Ugata, Y.; Yabuuchi, N. Activation of Anionic Redox for Stoichiometric and Li-Excess Metal Sulfides through Structural Disordering: Joint Experimental and Theoretical Study. *Journal of the American Chemical Society* 2025, *147* (30), 26238–26253. https://doi.org/10.1021/jacs.5c04018.

(38) Shiraiwa, T.; Yamasaki, T.; Kushimoto, K.; Kano, J.; Omata, T. Enhanced Proton Transport in Nb-Doped Rutile TiO2: A Highly Useful Class of Proton-Conducting Mixed Ionic Electronic Conductors. *Journal of the American Chemical Society* 2025, *147* (34), 30757–30767. https://doi.org/10.1021/jacs.5c05805.

(39) Ramesh, P.; Nithiananth, S.; Raman, K.; Annal Therese, H. Strategic Integration of Cobalt-Free Layered–Spinel Cathodes via Solution Combustion Method for High-Performance Lithium-Ion Batteries. *ACS Omega* 2025, *10* (46), 55669–55681. https://doi.org/10.1021/acsomega.5c06884.

(40) Mitchell, N. C.; Thomas, O. O.; Meyer, B. G.; Garcia-Fernandez, M.; Zhou, K.-J.; Grant, P. S.; Bruce, P. G.; Heap, R.; Sayers, R.; House, R. A. Influence of Ion Size on Structure and Redox Chemistry in Na-Rich and Li-Rich Disordered Rocksalt Battery Cathodes. *Advanced Materials* 2025, *37* (32), e2419878. https://doi.org/https://doi.org/10.1002/adma.202419878.

(41) Masoudi, M.; Xavier Jr, N. F.; Wright, J.; Roseveare, T. M.; Hinder, S.; Stolojan, V.; Cai, Q.; Slade, R. C. T.; Commandeur, D.; Gadkari, S. Ultralow Overpotential in Rechargeable Li–CO2 Batteries Enabled by Caesium Phosphomolybdate as an Effective Redox Catalyst. *Advanced Science* 2025, *12* (27), 2502553. https://doi.org/https://doi.org/10.1002/advs.202502553.

(42) Jing, P.; Inoishi, A.; Kobayashi, E.; Zhao, C.; Ren, P.; Abrahams, I.; Gregory, D. H. Self-Supporting Quasi-1D TaS3 Nanofiber Films with Dual Cationic/Anionic Redox for High-Performance Mg–Li Hybrid Ion Batteries. *ACS Applied Material & Interfaces* 2025, *17* (31), 44513–44527. https://doi.org/10.1021/acsami.5c09460.

(43) Hau, H.-M.; Holstun, T.; Lee, E.; Rinkel, B. L. D.; Mishra, T. P.; Markuson DiPrince, M.; Mohanakrishnan, R. S.; Self, E. C.; Persson, K. A.; McCloskey, B. D.; Ceder, G. Disordered Rocksalts as High-Energy and Earth-Abundant Li-Ion Cathodes. *Advanced Materials* 2025, *37* (46), e2502766. https://doi.org/https://doi.org/10.1002/adma.202502766.

(44) Kiran, A. S.; Shankar, E. G.; Nagaraju, M.; Su Yu, J. Binder-Free Phosphorus-Modified Multiphase Ni–Co Sulfide Nanoarchitectures with Sea-Urchin Morphology for High-Capacity Hybrid Supercapacitors and Practical Applications. *ChemSusChem* 2025, *18* (15), e202500588. https://doi.org/https://doi.org/10.1002/cssc.202500588.

(45) Stephens, I. D. R.; Parsons, A. C.; Burnett, D.; Slater, P.; Kendrick, E. Micro-Doping of Lithium-Ion Battery Cathode Materials - A Performance and Sustainability Case Study of Lithium Nickel

Oxide. *Global Challenges* 2025, *9* (10), e00345. https://doi.org/https://doi.org/10.1002/gch2.202500345.

(46) Chandrappa, S.; Forster-Tonigold, K.; a G, V.; Kannan, P.; Prakasha, K. R.; Gross, A.; Fichtner, M.; Caruso, R.; Karkera, G.; Annigere, P. Cobalt Borate Complex with Tetrahedrally Coordinated Co-Promotes Lithium Superoxide Formation in Li-O_2 Batteries. *Small* 2025, *21*. https://doi.org/10.1002/smll.202502150.

(47) Li, Y.; Zhao, J.; Zhang, S.; Fan, Y.; Kuo, C.-Y.; Ku, Y.-C.; Chan, T.-S.; Kao, C.-W.; Huang, Y.-C.; Chen, C.-T.; Haw, S.-C.; Jin, C.; Zhao, H.; Ye, D.; Jing, C.; Hu, Z.; Zhang, L. Hexavalent Ru Catalyst with Both Lattice Oxygen and Metal Ion Mechanisms Coactive for Water Oxidation. *Journal of the American Chemical Society* 2025, *147* (30), 26854–26864. https://doi.org/10.1021/jacs.5c08425.

(48) Baumgärtner, J. F.; Isler, D.; Nguyen, H. Q.; Klimpel, M.; Šivavec, J.; Černe, C.; Chernyshov, D.; van Beek, W.; Rettenwander, D.; Kravchyk, K. V; Kovalenko, M. V. A Highly Conductive Halospinel Cathode for All-Solid-State Batteries. *ACS Energy Letters* 2025, *10* (11), 5891–5899. https://doi.org/10.1021/acsenergylett.5c02476.

(49) Kong, S.; Matsui, N.; Hori, S.; Hirayama, M.; Mori, K.; Saito, T.; Kanno, R.; Suzuki, K. Exploration of Lithium-Ion Conductors Based on Local Coordination Environments Using Crystallographic Site Fingerprints. *Journal of the American Chemical ociety* 2025, *147* (28), 24336–24346. https://doi.org/10.1021/jacs.5c00856 .

(50) Rong, C.; Huang, X.; Arandiyan, H.; Shao, Z.; Wang, Y.; Chen, Y. Advances in Oxygen

Evolution Reaction Electrocatalysts via Direct Oxygen–Oxygen Radical Coupling Pathway. *Advanced Materials* 2025, *37* (9), e2416362. https://doi.org/https://doi.org/10.1002/adma.202416362.

(51) Nieto-Simón, J. A.; González-Barrios, M. M.; Gómez-Herrero, A.; Fernández-Díaz, M. T.; Prado-Gonjal, J.; Castillo-Martínez, E. Exploring Hollandite-Type KyVxTi8–XO16 (0.25 ≤ x ≤ 2) as Electrode Materials in Potassium-Ion Batteries (KIBs). *Inorganic Chemistry* 2025, *64* (17), 8578–8590. https://doi.org/10.1021/acs.inorgchem.4c05579.

(52) Parwaiz, S.; Khan, F.; Jennings, J. R.; Harunsani, M. H.; Kim, Y.-M.; Khan, M. M. Effect of Ni-Doping in LaCo1–XNixO3 on Electrocatalytic Oxygen Reduction Reaction. *ACS Omega* 2025, *10* (44), 52429–52441. https://doi.org/10.1021/acsomega.5c05357.

(53) Ghorbanzade, P.; Pesce, A.; Gomez, K.; López-Aranguren, P.; López Del Amo, J. M. Insights into the Compatibility and Interphases of Li6PS5Cl and Ga-Doped Li2ZrCl6 Halide Electrolytes by Solid-State NMR. *ACS Applied Energy Materials* 2025, *8* (15), 11205–11213. https://doi.org/10.1021/acsaem.5c01383.
